# Characteristics and Factors Associated with SARS-CoV-2 Infections in Individuals That Attended Referral Hospitals from Southern Region of Bahia State, Brazil: A Surveillance Network Retrospective Study

**DOI:** 10.3390/v13122462

**Published:** 2021-12-09

**Authors:** Fabrício Barbosa Ferreira, Galileu Barbosa Costa, Anaiá da Paixão Sevá, George Rego Albuquerque, Ana Paula Melo Mariano, Amanda Teixeira Sampaio Lopes, Hllytchaikra Ferraz Fehlberg, Íris Terezinha Santos de Santana, Pérola Rodrigues dos Santos, Luciano Cardoso Santos, Laine Lopes Silva de Jesus, Renato Fontana, Bianca Mendes Maciel, Mylene de Melo Silva, Luane Etienne Barreto, Sandra Rocha Gadelha

**Affiliations:** 1 Laboratório de Farmacogenômica e Epidemiologia Molecular, Departamento de Ciências Biológicas, Universidade Estadual de Santa Cruz, Ilhéus 45662-900, Bahia, Brazil; fabriciob.lisboa@hotmail.com (F.B.F.); gralbu@uesc.br (G.R.A.); apm.mariano@hotmail.com (A.P.M.M.); amanda_tsl@yahoo.com.br (A.T.S.L.); ferrazhellen@hotmail.com (H.F.F.); iristerezinha@gmail.com (Í.T.S.d.S.); prsantos.bio@uesc.br (P.R.d.S.); luciano.cardoso23@hotmail.com (L.C.S.); llsjesus.bio@uesc.br (L.L.S.d.J.); rfontana@uesc.br (R.F.); bmmaciel@uesc.br (B.M.M.); mmsilva@uesc.br (M.d.M.S.); luaneetienne@hotmail.com (L.E.B.); 2 Departamento de Ciências da Saúde, Universidade Estadual de Santa Cruz, Ilhéus 45662-900, Bahia, Brazil; 3 Departamento de Ciências Agrárias e AmBientais, Universidade Estadual de Santa Cruz, Ilhéus 45662-900, Bahia, Brazil; apseva@uesc.br; 4 Departamento de Ciências Biológicas, Universidade Estadual de Santa Cruz, Ilhéus 45662-900, Bahia, Brazil

**Keywords:** SARS-CoV-2, COVID-19, epidemiology, risk factors, healthcare professionals, Brazil

## Abstract

In December 2019, a novel coronavirus was detected in Wuhan, China, and rapidly spread worldwide. In Brazil, to date, there have been more than 20,000,000 confirmed cases of COVID-19 and more than 550,000 deaths. The purpose of the current study was to determine the clinical and epidemiological profile of the population affected by COVID-19 that have attended referral hospitals in Southern region of Bahia State, to better understand the disease and its risk factors in order to enable more appropriate conduct for patients. An observational, descriptive, cross-sectional, exploratory study was conducted using secondary data collected from the Laboratório de Farmacogenômica e Epidemiologia Molecular, Universidade Estadual de Santa Cruz (LAFEM/UESC). Chi-squared and Fisher’s exact tests were applied to determine the association between clinical symptoms and laboratory results, and to identify risk factors associated with SARS-CoV-2 infection. A total of 3135 individuals with suspected severe respiratory illness were analyzed and 41.4% of them tested positive for SARS-CoV-2 infection. Male individuals and having comorbidities were risk factors significantly associated with SARS-CoV-2 infection (OR = 1.17 and OR = 1.37, respectively). Interestingly, being a healthcare professional was a significantly protective factor (OR = 0.81, *p* < 0.001). Our findings highlight the importance of routinely testing the population for early identification of infected individuals, and also provide important information to health authorities and police makers to improve control measures, management, and screening protocols.

## 1. Introduction

The coronavirus disease 2019 (COVID-19), caused by the novel severe acute respiratory syndrome coronavirus 2 (SARS-CoV-2), emerged in Wuhan, China, in December 2019 [1]. It rapidly spread and received pandemic status from the World Health Organization (WHO) on 11 March 2020 [2,3]. In Brazil, the first officially registered COVID-19 case occurred on 26 February 2020 [4], and to date, there have already been more than 20,000,000 confirmed cases of COVID-19 in the country, with the number of deaths reaching more than 550,000 as of September 2021 [5].

Although most affected individuals may remain asymptomatic, the severity of COVID-19 can range from mild symptoms to severe illness [6]. The most common clinical symptoms are fever, cough, dyspnea, and myalgia [7], and there may also be diarrhea and sensory disorders, such as smell and taste [8,9,10]. It is important to point out that some of the affected individuals (10–20%) can progress to more critical outcomes, including severe pneumonia and respiratory failure, which requires hospitalization [7,11].

Individuals at risk of severe illness and death include elderly people and those with underlying conditions, such as hypertension, diabetes, obesity, chronic cardiovascular and respiratory disease, and cancer [6,7,11,12,13,14]. However, individuals from all age groups and with no history of comorbidities can also be severely affected [11,12,13,14,15]. Furthermore, males experience higher complications due to COVID-19 disease, although no differences in the proportion of males and females infected with SARS-CoV-2 has been observed [16].

During the pandemic, several preventive measures were put in place by public health authorities to track and contain the spread of SARS-CoV-2, and to care for those individuals presenting severe illness [17,18]. The opening of new clinical and Intensive Care Units (ICU), the implementation of field hospitals, as well as maintaining and enhancing the healthcare workforce to fight against the COVID-19 crisis are some examples. Indeed, healthcare professionals working on the front line are at high risk of infection, also being included in routine tests for early SARS-CoV-2 detection [19]. Furthermore, there have also been investments applied to increase the diagnostic capacity through new laboratories and complementary support by research laboratories from public universities [20].

Here, we aimed to characterize the clinical and epidemiological profile of individuals that attended different referral hospital networks from the Southern region of Bahia State, Brazil, also identifying the spectrum of risk factors for SARS-CoV-2 infection among front-line healthcare professionals. In addition, this study represents an opportunity to contribute to early identification of cases, avoiding or minimizing more severe outcomes related to COVID-19.

## 2. Materials and Methods

### 2.1. Study Design and Data Curation

An observational, descriptive, cross-sectional, exploratory study was conducted using secondary data from Laboratório de Farmacogenômica e Epidemiologia Molecular (LAFEM) database, located at Universidade Estadual de Santa Cruz (UESC). LAFEM/UESC works in partnership with the Central Public Health Laboratory Professor Gonçalo Moniz (LACEN–BA) and complementarily supports the routine diagnostics for SARS-CoV-2 detection in Southern region of Bahia State, which is one of the main epicenters of the COVID-19 pandemic after the capital Salvador and its metropolitan region.

During July to December 2020, a total of 3135 individuals with suspected severe respiratory illness were enrolled in this study, in which 541 were healthcare professionals. We included information from individuals that attended different referral hospitals in Southern region of Bahia State, which are Hospital Regional Costa do Cacau (HRCC) and Hospital de Ilhéus (HI), located in Ilhéus city (14°49′33.7″ S, 39°02′03.7″ W); Hospital Calixto Midlej Filho (HCM), and Hospital de Base Luis Eduardo Magalhães (HBLEM) located in Itabuna city (14°47′08″ S, 39°16′49″ W). Ilhéus and Itabuna are the third and the fifth largest cities in Bahia State, respectively, and reference centers in healthcare assistance for a large population located in the surroundings, as well as for leisure and commerce. Variables such as gender, age, self-reported skin color, clinical symptoms, comorbidities, and occupation were analyzed.

### 2.2. Laboratory Diagnosis

Nasopharyngeal swab specimens collected from study participants were tested at LAFEM/UESC. Viral RNA was obtained by using an automated Loccus EXTRACTA 32 device and a MVXA-P016 extraction kit. To detect SARS-CoV-2 RNA a RT-qPCR targeting regions of envelope (E), RNA-dependent RNA polymerase (RdRp), and nucleocapsid protein (N) genes were performed using the Allplex™ 2019-nCoV assay (Seegene^®^, Seoul, Korea) [21]. We also performed another RT-qPCR targeting N1 and N2 genes using the CDC 2019-Novel Coronavirus (2019-nCoV) Real-Time RT-PCR Diagnostic Panel [22], according to the manufacturer’s protocol.

### 2.3. COVID-19 Symptoms Assessment

A Chi-squared test with significance level of 5% (*p* ≤ 0.05) was applied to determine the association between the frequency of each reported clinical symptom and the results of RT-qPCR. To calculate the prevalence of clinical symptoms for different age groups, a 95% confidence interval was applied. The proportions of the agreements, positive and negative, between the clinical symptoms were calculated according to Cunningham and co-workers [23]. All analyses were performed using the R software version 3.6.1 [24].

### 2.4. Assessment of the Period of Symptoms until Laboratory Testing

Comparative analysis between the period from the onset of symptoms until the laboratory diagnosis (in individuals that tested positive or not for SARS-CoV-2) was performed using the Wilcoxon test after verifying the non-normality of data. A *p* value < 0.05 was considered to be significant. As COVID-19 is more severe in older individuals, a correlation analysis was performed between the period of symptoms until the laboratory tests and patient’s age. All analyses were performed using the R software version 3.6.1 [24].

### 2.5. Assessment of Factors Associated with SARS-CoV-2 Infection

To identify risk factors associated with SARS-CoV-2 infection, a bivariate analysis was carried out using Chi-squared and Fisher’s exact tests. Variables with a significance level of *p* < 0.20 were considered candidates to fit in the multivariate model, including all biologically plausible two-way interactions. A backwards approach was used and the best model was defined as the one that included significantly associated variables (*p* < 0.05) and minimized the value of the Akaike Information Criterion (AIC). Univariate analyzes were performed for variables that did not enter in the multivariate analysis. The proposed models were applied to all individuals. To minimize eventual bias, we also applied a separated model for healthcare professionals (HP) and the general public (excluding HP). Variables with less than 75% of the response rate and individuals with inconclusive results were excluded from the analysis. All analyses were performed using the R software version 3.6.1 [24].

### 2.6. Ethical Considerations

This study was submitted at Plataforma Brasil (a national and unified database of research records involving human beings for the entire Ethics in Research Committee/National Commission for Research Ethics, CEP/CONEP system) and approved by Research Ethics Committee of Universidade Estadual de Santa Cruz under registration number CAAE: 39142720.5.0000.5526.

## 3. Results

### 3.1. Demographic Characteristics of Study Population

During July to December 2020, a total of 3135 individuals with suspected COVID-19 underwent molecular detection of SARS-CoV-2 (Table 1). The majority of surveyed participants were female (54.3%) and the median age was 50 years (ranging from 1 to 103 years). Thirty-three samples (1.05%) had inconclusive results and were consequently removed from subsequent analyses. Among the samples that had satisfactory results (*n* = 3102), a total of 1285 (41.4%) tested positive for SARS-CoV-2 infection. Regarding the origin, most individuals (61.9%) were from Itabuna city. Only 21.5% of the individuals were from Ilhéus, and the remaining 26.5% were from the surrounding municipalities such as Canavieiras, Itajuípe, Uruçuca, Buerarema, Aurelino Leal, Itacaré, Camacan, Itapé, Coaraci, and Ibicaraí.

**Figure 1:**
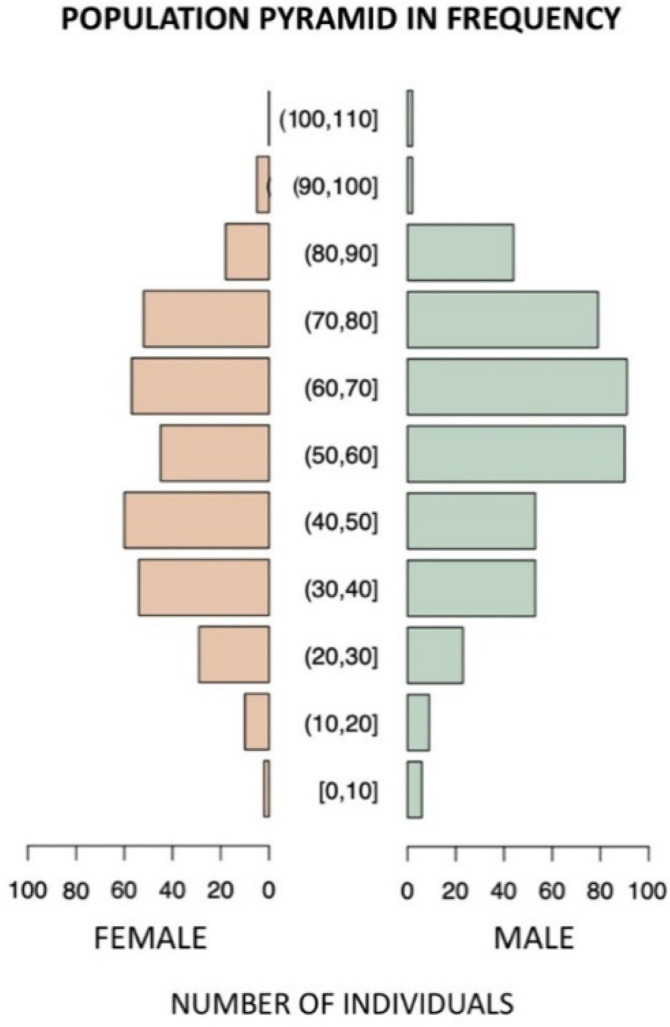
Frequency of individuals that tested positive for SARS-CoV-2 infection according to different age groups.

Figure 1 displays the frequency of individuals that tested positive for SARS-CoV-2. In general, male individuals were mostly affected, with a higher frequency among individuals aged 50–90 years. However, female individuals between 31 and 50 years were also highly affected.

### 3.2. Occupational Status of Study Participants

**Figure 2:**
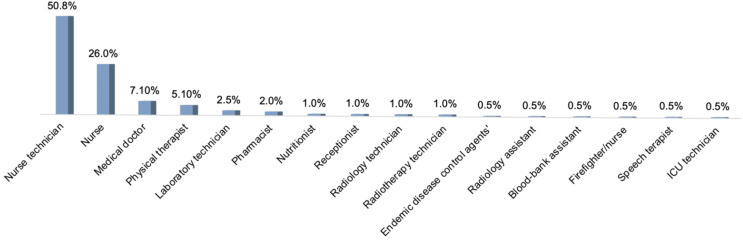
Frequency of reported occupational status of individuals that tested positive for SARS-CoV-2 infection.

For those individuals with satisfactory results of RT-qPCR, only 804 (25.9%) had information regarding their occupational status, in which 67.3% (541/804) were healthcare professionals. Among those healthcare professionals that tested positive for SARS-CoV-2, the highest frequency was observed in nurse technicians (including fellows or interns) (50.8%; *n* = 100) and nurses (26.0%; *n* = 51). Other healthcare professionals’ categories that tested positive for SARS-CoV-2 had frequencies lower than 10% (Figure 2).

### 3.3. Characterization of Clinical Symptoms

**Figure 3:**
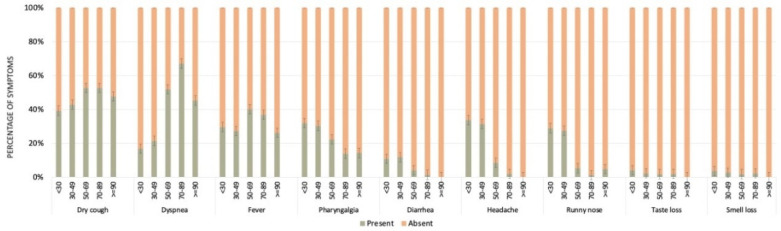
Proportion of individuals presenting the ten more frequent symptoms according to different age groups. Black dots represent the confidence intervals (high and low).

The most frequent symptoms reported by those individuals that tested positive for SARS-CoV-2 infection were fever, dyspnea, dry cough, and pharyngalgia (Figure 3). A comparative analysis between the main reported symptoms and results for SARS-CoV-2 infection is presented in Table 2. All symptoms were statistically different between individuals that tested positive and negative for SARS-CoV-2 infection. Failure to present or report all symptoms was more frequent in the negative group (*p* < 0.001).

Table 3 and Figure 3 show the frequencies of clinical symptoms according to different age groups. Fever was commonly reported among patients aged 50–69 years (50%; 95% Confidence Interval [CI]: 45.2–54.8%), and was significantly higher than individuals < 30 years, 30–49, and 70–89 years. On the other hand, dyspnea was more commonly reported among older patients, mainly aged 70–89 years (60.8%, CI: 55.2–66.4%), followed by patients aged 50–69 years (49.6%, CI: 44.8–54.4%), with a significant difference between them. Individuals aged 70–89 and 50–69 years also differed significantly from individuals aged < 30 and 30–49 years (15.4%; CI: 9.9–20.8% and 22.4%; CI: 18, 6–26.1%, respectively).

Dry cough was more common in individuals aged 50–69 years (63.0%, CI: 58.4–67.6%) and 70–89 years (59.9%, CI: 54.3–65.6%), with no significant difference between them. However, individuals aged 50–69 and 70–89 years old differed significantly from individuals aged <30 and 30–49 years (49.4%; CI: 41.8–57.0% and 50.3%; CI: 45.8–54.8%, respectively).

On the other hand, pharyngalgia was frequently reported by younger individuals (<30, 30–49, 50–69 years), but no significant differences were observed among them. Diarrhea was also frequently reported by younger individuals, aged 30–49 years (14.2%; CI: 11.0–17.3%), with a significant difference between individuals aged 50–69 years (5.0%; CI: 2.9–7.2%) and 70–89 years (2.4; CI: 0.7–4.2%). The frequency of headache and runny nose was very similar, being more frequent in individuals up to 49 years old, but no significant differences were observed. Similar results were observed for taste and smell losses, those symptoms being frequently reported by individuals up to 49 years old, with no significant differences observed.

### 3.4. Period of Onset of Clinical Symptoms until Laboratory Testing

The mean and median times from notification of symptoms to laboratory testing for those individuals that tested positive for SARS-CoV-2 were 6.75 and 5.00 days, respectively. For those individuals that tested negative for SARS-CoV-2, the mean and median times from notification of symptoms to laboratory testing were 6.37 and 4.00 days, respectively. The analysis of correlation between individuals’ age and the period from the onset of symptoms to laboratory testing was performed using the Spearman test (non-parametric). Although the *p* ≤ 0.001, the correlation was low (rho = 0.12 and R2 = 0.002), which cannot be considered significant.

### 3.5. Factors Significantly Associated with SARS-CoV-2 Infection

Statistical analysis revealed that healthcare professionals were less likely to test positive for SARS-CoV-2 (Odds Ratio [OR] = 0.81, *p* < 0.001) (Table 4). In order to verify the association between other specific variables, new analyses were performed separating the healthcare professional’s category from the remaining individuals.

Overall, individuals aged 50–89 years were more likely to test positive for SARS-CoV-2 (OR = 1.86 and 1.64, respectively) (Table 4), being a more representative and significant variable among the general population of the study (Table 5). Although healthcare professionals in these aged 50–69 and 70–89 years were more affected, no statistical significance was observed (Table 6).

Additionally, male individuals and having comorbidities were risk factors significantly associated with SARS-CoV-2 infection (OR = 1.17 and OR = 1.37, respectively) in the univariate analysis (Table 4). However, the same variables were not statistically significant when analyzed separately between the healthcare professionals’ group (Table 5) and the remaining individuals (Table 6).

## 4. Discussion

In this large study of adults attending referral hospitals with suspected SARS-CoV-2 infection, we showed that patients testing positive had substantial differences in gender, healthcare worker status, and the presence of comorbidities. As our study population was mostly composed of individuals with clinical suspicion for COVID-19 admitted at referral hospitals, or healthcare professionals working on the front line, high positivity for SARS-CoV-2 infection was expected. The 41.4% detection rate of SARS-CoV-2, therefore, does not reflect the rate of infection in the general population, but in hospitalized individuals. Our results were also higher compared to the results from a study conducted in the UK that found a prevalence of 33.4% [25].

We also showed that, on average, the time of positivity in the RT-qPCR test after the onset of symptoms was 6.75 days with a median of 5 days. Our results corroborate with the time frame recommended by health authorities [26,27] in which the laboratory tests should be performed 3–10 days after the onset of symptoms.

The most commonly reported symptoms were dry cough (41.4%), dyspnea (33.8%), and fever (28.9%), which corroborates studies conducted in Detroit [28] and New York [29] that found higher prevalence. A study performed in China showed that the frequency of fever was also higher, ranging from 43.8% in patients at the time of admission at the hospital to 88.7% during hospitalization [30]. Furthermore, it is important to emphasize that some studies have suggested screening clinical symptoms such as fever and/or dry cough as a criterion to perform laboratory tests. Thus, a higher frequency of clinical symptoms could be expected [29]. However, our study population were laboratory tested regardless the presence of specific symptoms.

Our study also demonstrated that the main symptoms associated with COVID-19 were different according to different age groups, in which greater illness severity and longer duration have been associated in older individuals. However, in a study carried out in China, only sore throat showed a significant difference between individuals aged younger and older than 60 years [30]. Furthermore, we noted that there was no specific symptom related to individuals testing positive or not for SARS-CoV-2, reinforcing the importance of laboratory diagnosis. Indeed, our findings highlight the importance of a differential diagnosis for coronavirus disease once most of symptoms are similar to other respiratory infections [7,8,10].

Regarding healthcare professionals, we found that nurses were more affected, corroborating other studies [31,32]. In fact, our data can be explained by long periods of direct contact with infected individuals, once nurses are one of the main healthcare professionals on the front line caring for patients with COVID-19 [31]. In addition, proportionally to other areas/professions, the nursing team is numerically the largest group of healthcare professionals in most hospitals.

It was found that individuals aged between 50 and 89 years were almost two times more likely to test positive for SARS-CoV-2 infection. Furthermore, male individuals and those with comorbidities were almost two times more likely to test positive for SARS-CoV-2 infection.

In our study, being a healthcare professional was a protective factor against SARS-CoV-2 infection (OR = 0.81, *p* < 0.001). A study conducted in Italy found that the profile of SARS-CoV-2 infection in COVID-19 units was almost the same as in other hospital units, suggesting that healthcare professionals working at COVID-19 units had a greater knowledge and risk perception compared to healthcare professionals at other hospital units [33]. One hypothesis that can help explain our findings is that healthcare professionals (mainly those working on the front line) have more and detailed access to health information, including prevention measures [33,34,35]. Additionally, healthcare professionals on the front line routinely use more effective protective personal equipment [36], and are more careful due to the constant exposure to individuals [33,34,35]. Furthermore, the advance of the pandemic has raised better perceptions of the risk among healthcare workers than the population in general, which could also help to explain our findings [34,35].

## 5. Conclusions

Data presented here are important to better understand the profile of SARS-CoV-2 infection within the hospital environment, provide insights into preventing future out-breaks of COVID-19, and contribute to mitigating the damage to healthcare systems. As most of patients evaluated here were asymptomatic, a comprehensive approach for symptom screening, routine testing, early identification, and contact tracing of more vulnerable individuals are important strategies to reduce transmission and stop the spread of the virus. This study also provides important and necessary information to health authorities and policymakers to improve control measures and management of the pandemic.

## Figures and Tables

**Table 1:** Frequency of analyzed individuals per hospital and according to RT-qPCR test for SARS-CoV-2 detection.

Hospital	Satisfactory Results		Total	Inconclusive
	Positive (%)	Negative (%)		
Hospital de Base Luis Eduardo Magalhães (HBLEM)	296 (30.4)	677 (69.6)	973	14
Hospital Calixto Midlej Filho (HCM)	371 (43.6)	479 (56.4)	850	6
Hospital de Ilhéus (HI)	142 (41.2)	203 (58.8)	345	1
Hospital Regional Costa do Cacau (HRCC)	476 (50.9)	458 (49.1)	934	12

**Table 2:** Frequency of reported symptoms among individuals tested for SARS-CoV-2 infection.

Symptoms	RT-qPCR for SARS-CoV-2		Qui-Squared Test		
	Positive (%)	Negative (%)	χ^2^	df	*p* Value
Fever Yes No	580 (54.3) 787 (28.2)	488 (45.7) 2007 (71.8)	230.8	1	<0.001
Dyspnea Yes No	524 (47.4) 848 (30.6)	581 (52.6) 1924 (69.4)	98.4	1	<0.001
Dry cough Yes No	772 (50.7) 600 (25.5)	752 (49.3) 1754 (74.5)	256.9	1	<0.001
Pharyngalgia Yes No	392 (45.5) 976 (32.4)	469 (54.5) 2032 (67.6)	49.9	1	<0.001
Diarrhea Yes No	110 (44.9) 1254 (34.7)	135 (55.1) 2355 (65.3)	10.89	1	<0.001
Headache Yes No	279 (40.6) 1083 (34.2)	409 (59.4) 2083 (65.8)	9.6	1	<0.001
Runny nose Yes No	256 (43.5) 1109 (33.9)	332 (56.5) 2158 (66.1)	19.5	1	<0.001
Taste loss Yes No	78 (64.5) 1286 (34.5)	43 (35.5) 2446 (65.5)	46.1	1	<0.001
Smell loss Yes No	83 (65.4) 1280 (34.4)	44 (34.6) 2445 (65.6)	46.1	1	<0.001

χ^2^: Chi-squared; df: degree of freedom.

**Table 3:** *p* values of Chi-squared calculation between clinical symptom and age groups paired.

Symptoms	Age Groups	Age Groups			
		<30	30–49	50–69	70–89
Fever	30–49	1.000			
	50–69	**0.014**	**0.001**		
	70–89	0.800	0.745	**0.011**	
	≥90	0.621	0.614	1.000	0.695
Dyspnea	30–49	0.069			
	50–69	**0.000**	**0.000**		
	70–89	**0.000**	**0.000**	**0.004**	
	≥90	**0.008**	0.058	1.000	0.653
Dry cough	30–49	0.910			
	50–69	**0.003**	**0.000**		
	70–89	**0.036**	**0.012**	0.452	
	≥90	0.392	0.408	1.000	0.868
Pharyngalgia	30–49	0.509			
	50–69	0.128	0.269		
	70–89	**0.001**	**0.001**	**0.033**	
	≥90	0.701	0.856	1.000	1.000
Diarrhea	30–49	0.065			
	50–69	0.192	**0.000**		
	70–89	**0.008**	**0.000**	0.123	
	≥90	0.632	0.327	0.904	1.000
Headache	30–49	1.000			
	50–69	**0.000**	**0.000**		
	70–89	**0.000**	**0.000**	**0.001**	
	≥90	**0.027**	**0.024**	0.532	1.000
Runny nose	30–49	0.932			
	50–69	**0.000**	**0.000**		
	70–89	**0.000**	**0.000**	**0.000**	
	≥90	**0.044**	**0.037**	0.559	1.000
Taste loss	30–49	0.110			
	50–69	**0.000**	**0.010**		
	70–89	**0.000**	**0.000**	0.120	
	≥90	0.405	0.640	1.000	1.000
Smell loss	30–49	0.582			
	50–69	**0.000**	**0.000**		
	70–89	**0.000**	**0.000**	0.549	
	≥90	0.459	0.545	1.000	1.000

The bold highlights the significant *p*-value.

**Table 4:** Characteristics associated with SARS-CoV-2 infection in all individuals (*n* = 3102) that attended referral hospitals in Southern Bahia State, Brazil.

Variables	RT-qPCR for SARS-CoV-2			Multivariate Model		Univariate Analysis	
	Total Individuals	Positive (%)	Negative (%)	OR (95% CI)	*p* Value	OR (95% CI)	*p* Value
Gender * Female Male	1685 1417	668 (39.6) 617 (43.5)	1017 (60.4) 800 (56.5)			Reference 1.17 (1.02–1.36)	0.028
Age group <30 30–49 50–69 70–89 ≥90 Not informed **	401 1223 824 604 48 2	128 (31.9) 443 (36.2) 404 (49.0) 294 (48.7) 14 (29.2)	273 (68.1) 780 (63.8) 420 (51.0) 310 (51.3) 34 (70.8)	Reference 1.22 (0.94–1.57) 1.86 (1.42–2.44) 1.64 (1.23–2.19) 0.91 (0.46–1.78)	0.124 <0.001 <0.001 0.783		
Self-reported skin color ^‡^ Mixed White/Yellow/Indigen Black Not informed **	849 337 127 1789	381 (44.9) 156 (42.0) 49 (38.6)	468 (55.1) 181 (58.0) 78 (61.4)			Reference 1.05 (0.82–1.36) 0.77 (0.53–1.13)	0.659 0.183
Comorbidities * NoYes	2282 820	899 (39.4) 386 (47.1)	1383 (60.6) 434 (52.9)			Reference 1.37 (1.17–1.61)	<0.001
Healthcare professionals No Yes Not informed **	1886 833 383	802 (42.5) 281 (33.7)	1084 (57.5) 552 (66.3)	Reference 0.81 (0.67–0.99)	<0.001		

OR: Odds Ratio; CI: Confidence Interval. * Variables were not included in the multivariate model. ** Not included in statistical analysis. ^‡^ Not included in statistical analysis due to the high number of individuals without information.

**Table 5:** Characteristics associated with SARS-CoV-2 infection in individuals (n = 1886) that attended referral hospitals in Southern Bahia State, Brazil.

Variables	RT-qPCR for SARS-CoV-2			Multivariate Model		Univariate Analysis	
	Total Individuals	Positive (%)	Negative (%)	OR (95% CI)	*p* Value	OR (95% CI)	*p* Value
Gender * Female Male	855 1031	355 (41.5) 447 (43.4)	500 (58.5) 584 (56.6)			Reference 1.08 (0.9–1.29)	0.436
Age group <30 30–49 50–69 70–89 ≥90 Not informed **	221 512 599 510 43 1	73 (33.0) 192 (37.5) 293 (48.9) 229 (44.9) 14 (32.6)	148 (67.0) 320 (62.5) 306 (51.1) 281 (55.1) 29 (67.4)	Reference 1.22 (0.87–1.69) 1.94 (1.4–2.68) 1.65 (1.19–2.29) 0.98 (0.48–1.96)	0.248 <0.001 <0.001 0.095		
Self-reported skin color ^‡^ Mixed White/Yellow/Indigen Black Not informed **	706 274 93 813	326 (46.2) 131 (47.8) 43 (46.2)	380 (53.8) 143 (52.2) 50 (53.8)			Reference 1.07 (0.81–1.41) 1.00 (0.65–1.55)	0.645 0.991
Comorbidities * No Yes	1265 621	527 (41.7) 275 (44.3)	738 (58.3) 346 (55.7)			Reference 1.11 (0.92–1.35)	0.279

OR: Odds Ratio; CI: Confidence Interval * Variables were not included in the multivariate model. ** Not included in statistical analysis. ^‡^ Not included in statistical analysis due to the high number of individuals without information.

**Table 6:** Characteristics associated with SARS-CoV-2 infection in healthcare professionals (n = 833) that attended referral hospitals in Southern Bahia State, Brazil.

Variables	RT-qPCR for SARS-CoV-2			Multivariate Model		Univariate Analysis	
	Total Individuals	Positive (%)	Negative (%)	OR (95% CI)	*p* Value	OR (95% CI)	*p* Value
Gender Female Male	637 196	224 (35.2) 57 (29.1)	413 (64.8) 139 (70.9)	Reference 0.76 (0.53–1.07)	0.112		
Age group * <30 30–49 50–69 70–89 ≥90 Not informed **	142 593 92 4 2 0	42 (29.6) 200 (33.7) 36 (39.1) 3 (75.0) 0	100 (70.4) 393 (66.3) 56 (60.9) 1 (25.0) 2 (100.0)	Reference 1.22 (0.87–1.69) 1.94 (1.4–2.68) 1.65 (1.19–2.29) 0.98 (0.48–1.96)	0.248 <0.001 <0.001 0.095	Reference 1.21 (0.81–1.80) 1.53 (0.88–2.66) 0.14(0.72–70.65) 0 (0-Inf)	0.345 0.131 0.093 0.97
Self-reported skin color ^‡^ Mixed White/Yellow/Indigen Black Not informed **	83 39 31 680	23 (27.7) 14 (35.9) 3 (9.7)	60 (72.3) 25 (64.1) 28 (90.3)			Reference 1.46 (0.65–3.29) 0.28 (0.08–1.01)	0.36 0.052
Comorbidities * No Yes	774 59	257 (33.2) 24 (40.7)	517 (66.8) 35 (59.3)			Reference 1.38 (0.8–2.37)	0.248

OR: Odds Ratio; CI: Confidence Interval. * Variables were not included in the multivariate model. ** Not included in statistical analysis. ^‡^ Not included in statistical analysis due to the high number of individuals without information.

## Data Availability

Not applicable.
